# Quality over quantity: how to get the best results when using docking for repurposing

**DOI:** 10.3389/fbinf.2025.1536504

**Published:** 2025-05-26

**Authors:** Lenin Domínguez-Ramírez, Maricruz Anaya-Ruiz, Paulina Cortés-Hernández

**Affiliations:** ^1^ Computational Biochemistry Laboratory, Centro de Investigación Biomédica de Oriente (CIBIOR), Instituto Mexicano del Seguro Social (IMSS), Atlixco, Mexico; ^2^ Cancer Cell Biology, Centro de Investigación Biomédica de Oriente (CIBIOR), Instituto Mexicano del Seguro Social (IMSS), Atlixco, Mexico

**Keywords:** docking, GNINA, drug repurposing, virtual screening, convolutional neural network, UCSF ZINC, Dock6, AutoDock Vina

## Abstract

Molecular docking is among the fastest and most readily available computational tools to explore protein–ligand interactions. However, little effort has been put into assessing the quality of its results. In this paper, we compared eight free license docking programs to screen a drug library against the human target, phosphodiesterase 5A (PDE5A), to evaluate their ability to find its known ligand, sildenafil, and other ligands that became erectile dysfunction drugs because they inhibit this target. GNINA was superior at identifying the known target because it offers a convolutional neural network (CNN) score that ranks the quality of docking results. Using this CNN score improved the ranking of known positives. Receiver operating characteristic (ROC) analysis revealed that all docking suites lack specificity; that is, they often misidentify true negatives. Employing a CNN score cutoff before ranking by docking affinity raised specificity with a small loss in sensitivity. After the cutoff, datasets became smaller but of higher quality. We propose a heuristic to produce relevant docking results, which includes an overall evaluation of the target on docking performance through ROC and an improvement of candidate binder selection using a CNN score cutoff of 0.9.

## Introduction

Molecular docking is an ever-growing field of techniques aimed at rapid exploration of molecular binding interactions to be analyzed with other *in silico* (molecular dynamics, alchemical, ADMET, and quantum mechanics) (Kumar et al., 2024) and *in vitro* (high-throughput screening versus cellular targets) (Drayman et al., 2021; Chunarkar-Patil et al., 2024) methods. Search algorithms and scoring functions have evolved, transitioning from empirical pose evaluation to machine learning, convolutional neural networks, and generative models (Crampon et al., 2022). Currently, docking packages with free or paid licenses use different search algorithms, multiple scoring methods, and varied hardware. Most run in Unix-based operating systems. Some require GPUs (McNutt et al., 2021; Corso et al., 2022; Solis-Vasquez et al., 2022), and most benefit from high CPUs. RAM or disk space requirements are moderate. Computer clusters are only required when screening millions of compounds. GNINA is a newer docking program that uses Vina scoring functions as well as its own, based on convolutional neural networks (CNN), performing better than Vina, even when water molecules are not explicitly modeled (Hochuli et al., 2018; Francoeur et al., 2020).

As a fast and inexpensive tool to rank potential binders, docking retains limitations. First, it is based on the “lock and key” hypothesis (Ehrlich, 1907), where receptor and ligand are rigid bodies (Ferro and Hermans, 1977; Meng et al., 2011). Ligand flexibility can be modeled at a computational cost but without an exhaustive degree of freedom exploration. Second, the energetics calculated with docking are inaccurate (at least ±3 kcal from experimentally determined values). Third, there is a limitation on the volume that can be sampled: as grid sizes increase, sampling becomes inefficient. Fourth, the systematic evaluation of docking results has not been solved, and this represents a large gap in the field. Here, we approach this fourth limitation: evaluation of result quality.

Instead of following a common set of criteria, docking performance is evaluated with relative parameters put together on a case-by-case basis. These parameters usually entail affinity ranking of the poses and/or structural comparisons to known experimentally determined complexes and/or redocking, which limits their applicability to *de novo* complexes without reference. None of these parameters has a universal accuracy threshold. Any given docking experiment will return a binding affinity value even if interactions are unspecific, so ranking based only on the affinity does not guarantee accuracy. If a docked complex displays a 2 Å root mean square deviation (RMSD) difference or less to an experimental complex, docking is usually considered accurate (Zajaček et al., 2024), but there are no guidelines to distinguish true positive binders from high affinity negatives, or to define a threshold that is useful to detect leads to explore further. It is also unclear what quality control criteria should be included in all docking publications or how a user should choose a docking program for a specific target.

Receiver operating characteristic (ROC) analysis (Mandrekar, 2010) allows the comparison of binary classifiers. In the case of docking, ROC characterizes the ability of each method (docking software) to distinguish between true and false binders, using the binding energy as the classifying value. The area under the curve (AUC) of a ROC analysis lets the user of a method distinguish between a good classifier (AUC ≥0.70) and one that is closer to a random guess (AUC ≤0.5) and has been used by developers to evaluate their docking results. RxDock reported AUC results for their method (Ruiz-Carmona et al., 2014), and UCSF DOCK publishes ROC curves for representative targets (Brozell et al., 2012; Allen et al., 2015). The ROC plot displays specificity on the x-axis (false positive rate) and sensitivity (true positive rate) on the y-axis and calculates true negative and false negative rates. Allen et al. (2015) exemplify receptors with good and bad curves. However, most docking publications do not show an ROC analysis, making evaluating results beyond the affinities presented difficult.

The current work explores how to discriminate true positives and grade the quality of docking results. We compared eight docking suites available with free licenses and attempted to identify drugs for erectile dysfunction using human phosphodiesterase-5 (PDE5A). PDE5A hydrolyzes cyclic GMP (cGMP) to 5’-GMP, and it is the target of the erectile dysfunction drug sildenafil, which acts as a competitive inhibitor for cGMP degradation (Sung et al., 2003; Zhang et al., 2004). Sildenafil was originally developed for pulmonary hypertension; during clinical testing, it produced penile erections through PDE5A inhibition and was soon commercialized under the name Viagra. It is a famous example of drug repurposing. We asked whether current free docking packages can select binders that inhibit PDE5A from a database of FDA-approved drugs that includes sildenafil and other compounds developed for this very target. We evaluated the quality of the results with ROC analysis and with the CNN score from GNINA. The CNN score was the best evaluator of docking result quality to select true positives on a virtual screen.

## Materials and methods

### Software

We evaluated eight docking programs available on a free license, so our work can be reproduced without monetary expense: ADFR, v. 1.1 (Ravindranath et al., 2015), AutoDock Vina, v1.1.2 (Trott and Olson, 2010), UCSF DOCK, v6.9 (Allen et al., 2015), GNINA, v1.0.3 (McNutt et al., 2021), jdock, v2.2.3c (Li et al., 2012), PLANTS, v1.2 (Korb et al., 2009), RxDock, v0.1.0 (Ruiz-Carmona et al., 2014), and smina, v1.1.2 (Koes et al., 2013). The selection includes the two most widely used packages: UCSF DOCK and AutoDock Vina. UCSF DOCK and RxDock were downloaded as source code and compiled locally. The other programs are available as precompiled executables. ADFR, AutoDock Vina, jdock, smina, and RxDock return an affinity in kcal/mol; UCSF DOCK gives a gridscore, while GNINA returns three values: GNINA affinity in kcal/mol, an adimensional CNN score (0–1), and a CNN affinity [−log (K)].

### Receptor selection and preparation for the docking screen

The binding target was the human cGMP-specific 3′,5′-cyclic phosphodiesterase (PDE5A, UNIPROT ID: O76074) (Zhang et al., 2004), a metalloenzyme containing zinc and magnesium. There are 38 X-ray diffraction (XRD) PDE5A structures deposited in RCSB, four crystalized with sildenafil (PDBID: 2H42, 1UDT, 3JWQ, and 1TBF). An AlphaFold model was obtained using the PDE5A sequence from UNIPROT through UCSF ChimeraX, v1.7. All 38 PDE5A structures and the AlphaFold model were evaluated, and structures 1T9R, 1T9S, and the AlphaFold model were tested in a screen against a ligand database.

Because ADFR, Vina, GNINA, jdock, and smina use pdbqt receptors and ligands, both sets were prepared using the scripts included with ADFR (prepare_receptor.py and prepare_ligand.py). UCSF DOCK and PLANTS require mol2 files; receptors for UCSF DOCK were prepared with UCSF Chimera (Pettersen et al., 2004). For PLANTS, receptors were processed with SPORES, v1.3 (Brink and Exner, 2010).

### Ligand preparation for a docking screen

Ligands were obtained from UCSF ZINC 12: a total of 2115 FDA-approved drugs, including stereoisomers (Irwin et al., 2012), were screened, that is, they were all docked to each of our three receptors (1T9R, 1T9S, and the AlphaFold model). Other than conversion to mol2 or pdbqt, no other preprocessing was performed. PLANTS failed with four ligands; jdock failed with 41, and ADFR failed with one. Failure reasons were not identified or corrected. A total of 513 experimentally determined binders for PDE5A were obtained from UCSF ZINC by selecting the results with affinities better than 10 nM. These were used as true positive results for ROC calculation.

Ligands were converted into mol2 using Racoon, v1.0f (Forli et al., 2016). RxDock requires sd files for ligands; these were prepared with Open Babel, v3.1.0 (O’Boyle et al., 2011), while the receptor is a mol2 file prepared with UCSF Chimera, v alpha 1.19. ADFR requires an additional step to create a .trg file.

File formats for the ligands and receptors, as well as instructions to prepare them, are summarized in Supplementary Table S1.

### Docking parameters

The coordinates and size of the search were defined using the largest cavity in the PDE5A models, found by AGFGRUI and used for all docking programs except UCSF DOCK and RxDock. For Dock and RxDock, the search box was generated using GMP (called 5 GP in the PDB) as a reference. Search coordinates were the same for all systems, and grid sizes are specified in Supplementary Table S2. All programs were run with default values for the number of results and other parameters (see supplemental material Command Line for specific details). Running time for docking ranged from 4 h to 18 h for each program. Only the enhanced searches (using ADFR, Vina, and GNINA) required longer running times. All docking was conducted without Zn because only PLANTS can handle Zn atoms. All ligands and water molecules in XRD structures were removed before docking experiments.

ADFR, Vina, and PLANTS were executed in the “Laboratorio Nacional de Supercómputo del Sureste de Mexico” facility; GNINA, jdock, RxDock, smina, and UCSF DOCK were run on a workstation under Debian 11. An RTX 3060 GPU was used for GNINA. The most recent GNINA version, v1.3, can be run on CPUs.

### Analysis

Most docking programs return several results per input ligand. For analysis, we used only the one with the highest binding energy.

ROC calculations were performed using R (R Core Team, 2021) and RStudio (RStudio, 2020) using libraries pROC (Robin et al., 2011) and cutpointr (Thiele and Hirschfeld, 2021). All relevant files (initial files and results) are available at figshare with doi: 10.6084/m9.figshare.24217872. Ligand RMSD was calculated using DockRMSD (Bell and Zhang, 2019).

## Results

### PDE5A structures compared through ensemble docking

**FIGURE 1:**
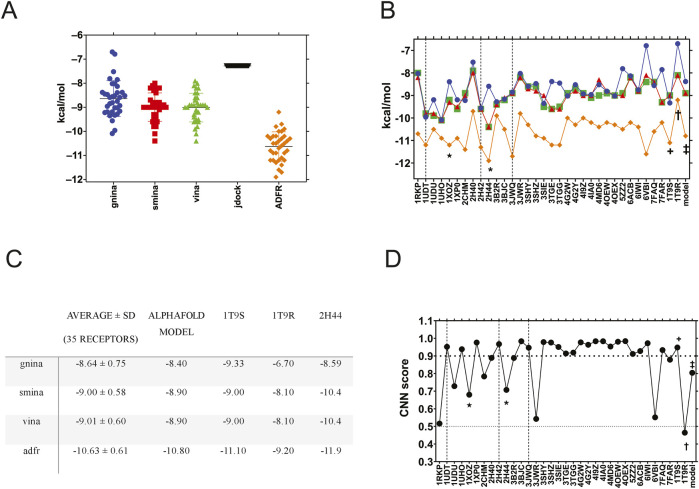
Ensemble docking of sildenafil against 35 human PDE5A structures. **(A)** Sildenafil binding affinity by docking software, with all the PDE5A structures grouped to compare between the docking packages. **(B)** Sildenafil docking results for GNINA (blue circles), smina (red squares), Vina (green triangles), and ADFR (orange diamonds) are displayed by each of the 35 PDE5A receptors (on the x-axis). **(C)** Average affinity of the ensemble (35 receptors) compared to that of the specific structures used. **(D)** Docking results were evaluated using the CNN score from GNINA. The horizontal bold dotted line indicates a 0.9 cutoff, while the dotted line indicates a 0.5 cutoff. Dashed vertical lines indicate structures crystallized with sildenafil, while * indicates a structure with icarisid II, and ★ indicates a structure with tadalafil. The receptors used in this work are labeled +, †, and ‡ for 1T9S, 1T9R, and the AlphaFold model, respectively.

First, we compared the 38 available PDE5A XRD structures and the AlphaFold model through ensemble docking. Four structures could not be used (1TBF, 3HC8, 3HDZ, and 6L6E) because they were not successfully converted to pdbqt or otherwise processed through *agfrgui*; this was due to sidechains with multiple occupancies. No corrections were attempted due to a lack of a clear heuristic, resulting in 35 target structures. Ensemble docking with sildenafil was performed for these structures with the docking packages that allowed it (Figure 1A). UCSF DOCK, PLANTS, and RxDock were excluded due to command line limitations. ADFR produced the highest affinities, followed by Vina, smina, and GNINA, with similar performance, which was expected because the latter two are based on Vina. jdock returned the same affinity for sildenafil on every receptor, suggesting that it is not suitable for ensemble docking (Figure 1A). The ensemble experiment results are shown in Figure 1B, graphed by the receptor used. The figure shows that receptor selection impacts docking results. Structures displayed different binding affinities for sildenafil, with a small standard deviation of 0.58–0.75 kcal/mol (Figure 1C). The best affinity was for receptor 2H44 (* in Figure 1B), a structure determined in the presence of icarisid II, that displayed high affinity when using smina, Vina, and ADFR (Figures 1B,C). The three structures crystallized with sildenafil (vertical lines) also showed high affinities for sildenafil (Figures 1B,C).

### PDE5A structure selection for a virtual docking screen

To perform a virtual screen against all the FDA-approved drugs (screen described in the next section), we selected three PDE5A structures that represent the conformational diversity of the binding site: 1T9S, a chimeric structure crystalized with the enzyme’s catalytic product GMP that could bind inhibitors like sildenafil and similar molecules; 1T9R crystallized with an occluded, empty binding site; and the AlphaFold model that we created for PDE5A to simulate a docking study where targets have no previous crystallographic reference. 1T9R was the most different, with an RMSD of ∼3.5 Å compared to the other two (Supplemental Table 3) due to its “H-loop” (residues L672-H685), located over the GMP binding site (Zhang et al., 2004). We did not select a structure crystallized with sildenafil because most docking campaigns for repurposing would not have such a reference available. In the ensemble experiment, GNINA, smina, Vina, and ADFR docked sildenafil to 1T9S (†) and to the AlphaFold model (‡) with affinities close to the ensemble average (Figure 1C). 1T9R, with a closed H-loop over the binding site, displayed less affinity on all the programs evaluated (Figures 1B,C).

GNINA calculates not only a “GNINA affinity” but also a CNN score, which grades the quality of docking results through network-trained image recognition algorithms. GNINA also produces a second affinity, based on the CNN model (“CNN affinity”). In Figure 1D, we graphed each receptor against its CNN score for sildenafil from the ensemble docking in GNINA. The three structures crystalized with sildenafil (vertical lines) had higher scores than an arbitrary CNN score cutoff of 0.9 that we hereby defined as “high-quality docking.” 1T9S also scored as high quality, but the CNN score was lower than this threshold for the AlphaFold model and 11 other structures. Visual examination of receptors below this 0.9 CNN score cutoff reveals receptors with occluded binding sites or missing residues. The worst-scoring was 1T9R, likely due to its occluded binding site. These results reaffirm our selection of 1T9S, 1T9R, and the AlphaFold model to represent the conformational diversity of PDE5A’s binding site.

### Docking screen to three PDE5A structures and result evaluation by rank

**FIGURE 2:**
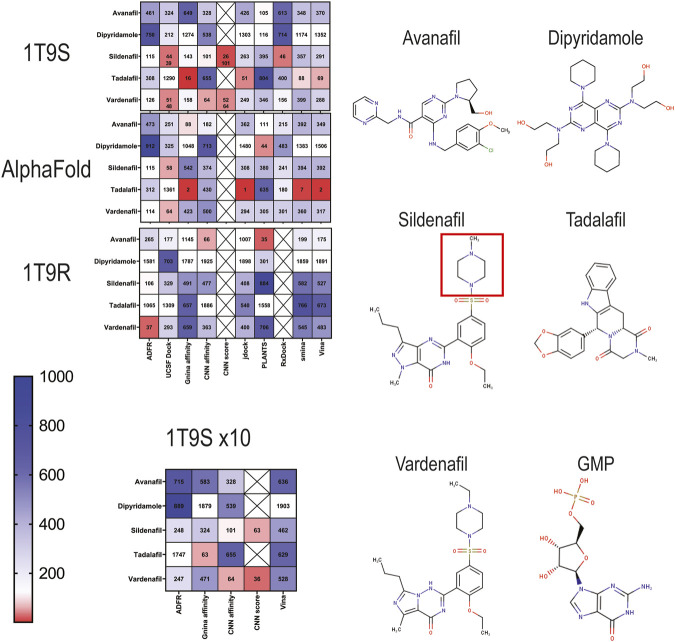
Ranking of docking results of known PDE5A inhibitors into three PDE5A models (1T9S, AlphaFold model, and 1T9R) represented as heatmaps scaled from red (highest rank) to blue (lowest rank) among 2115 FDA-approved drugs. Crossed squares indicate that the data are not available. 1T9S x10 indicates that docking was conducted at high exhaustiveness. The structures for the drugs that target PDE5A are shown. The red square is the ethylpiperazine motif.

Next, we docked a dataset of 2,115 approved drugs on the three selected PDE5A structures (1T9S, AlphaFold model, and 1T9R) using each of the eight docking packages. We ranked results by affinity from highest to lowest, then looked for the known PDE5A inhibitors: sildenafil and vardenafil, which are chemically similar, sharing a guanosine-like core with GMP, and tadalafil, avanafil, and dipyridamole, which diverge structurally (Figure 2). The ordinal position of these inhibitors in the ranking was compared (Figure 2). For 1T9S, UCSF DOCK, and RxDock detected sildenafil in the 44th and 46th place (Figure 2, 1T9S). CNN affinity picked sildenafil at rank 101, better than rank 143 by GNINA’s affinity. In addition, vardenafil and tadalafil were ranked in the top 100 best results by several packages. Tadalafil was 16th by GNINA, 51st by jdock, 69th by Vina, and 88th by smina. Vardenafil was 51st by UCSF DOCK and 64th by CNN affinity. Cartesian minimization after docking with UCSF DOCK improved sildenafil’s rank to 39 and vardenafil’s to 48.

When the AlphaFold model was used as a receptor, UCSF DOCK identified sildenafil as the 58th best ligand. No other program ranked it better than 115th. Tadalafil ranked remarkably well, first by JDock, second by GNINA and Vina, and seventh by smina, while vardenafil was 64th with UCSF DOCK (Figure 2, AlphaFold). CNN affinity did not improve the ranking of known ligands in the AlphaFold model.

When docking to 1T9R (closed binding site), the best ranking for sildenafil was 106, with ADFR (Figure 2, 1T9R). ADFR picked vardenafil in place 37. Avanafil was 66th by CNN Affinity and 35th by PLANTS. As expected, 1T9R did not find sildenafil in the top ligands, and known PDE5A ligands consistently ranked worse than in the other two target structures.

Up to this point, all programs had been executed using moderate screening settings for exhaustiveness and number of runs. For 1T9S, an additional set of dockings was produced with a ten-fold increase in runs at high exhaustiveness, with the three programs that supported it, ADFR, GNINA, and Vina (Figure 2, 1T9S x10). Generally, rankings for the known PDE5A inhibitors were worse with more exhaustiveness, while CNN affinity remained unaffected. Thus, more computational power and time did not improve the identification of known binders.

### CNN score to screen the best poses

We used the CNN score produced by GNINA to extract the best poses according to the arbitrary ≥0.90 threshold for high-quality results presented in Figure 1D. Only the poses that surpassed this quality threshold were selected and then ranked by regular GNINA affinity or by CNN affinity. This heuristic, incorporating the CNN quality score, did not find sildenafil or any other known PDE5A inhibitors as a hit for the AlphaFold model or for 1T9R (Figure 2, CNN score). In contrast, for 1T9S, it ranked sildenafil and vardenafil better than when using docking affinity: sildenafil was 26th and 101st, while vardenafil was 52nd and 64th using GNINA’s affinity and CNN affinity, respectively (Figure 2, see row: “CNN score”). Figure 1D shows that the CNN score reveals a useful distinction between receptors with poor (1T9R) and high-quality (1T9S) binding sites. Our AlphaFold model is closer to high quality, yet critically below the 0.9 CNN score threshold. Thus, when a ligand that is a known true positive (like sildenafil) is available to evaluate a receptor, the CNN score is a useful metric to select a receptor to find other true positives. Once a good receptor is selected, the CNN score can be used to further filter docking screening results, improving their ranking.

### Docking result evaluation through ROC curves

Receiver operating characteristic (ROC) curves have been used to evaluate docking performance, so we used them to compare the three target structures and eight docking programs analyzed here. To create the ROC analyses, we docked 513 experimentally determined binders (all curves are shown in Supplementary Figure S1) and the set of 2,115 FDA-approved drugs into each structure (1T9S, AlphaFold model and 1T9R) with each program (eight suites plus the CNN affinity from GNINA) resulting in 27 initial ROC curves (Table 1). Overall, the AUC values and Youden’s index were better for 1T9S (Table 1). The best AUC values were found with CNN affinity, PLANTS, and ADFR for all targets. Accuracy, precision, and sensitivity displayed acceptable performance across all programs. However, specificity, that is, the portion of true negatives correctly identified, was low (under 0.5) except when using CNN affinity. Cutoff points represent the best method of distinguishing true positives from true negatives, and they were higher for 1T9S, but values are dependent on the scale used by each docking package and were thus not directly comparable between them. CNN affinity cutoff values are positive because they correspond to −log(K).

**TABLE 1:** ROC analysis for docking using 1T9S, the AlphaFold model, and 1T9R.

	AUC (95% CI)	Accuracy	Precision	Sensitivity	Specificity	Youden’s index	Cutoff point
1T9S							
ADFR	0.85 (0.83–0.89)	0.82	0.85	0.93	0.39	0.57	−9.3
DOCK	0.73 (0.70–0.77)	0.71	0.74	0.95	0.02	0.43	−64.98
	0.67 (0.66–0.73)*	0.72*	0.74*	0.96*	0.00	0.34	−47.34
GNINA	0.78 (0.74–0.80)	0.79	0.81	0.95	0.15	0.43	−8.1
	0.84† (0.78–0.93)	0.90	0.95	0.82	0.97	0.66	−8.1
CNN affinity+	0.89 (0.88–0.92)	0.86	0.88	0.95	0.53	0.67	6.89
	0.96† (0.94–1)	0.92†	0.95†	0.92†	0.92†	0.84†	7.05†
JDOCK	0.80 (0.77–0.83)	0.81	0.84	0.95	0.25	0.47	−8.82
PLANTS	0.87 (0.85–0.90)	0.81	0.85	0.92	0.48	0.6	−91.25
RXDOCK	0.67 (0.60–0.67)	0.71	0.72	0.98	0.11	0.3	−17.97
SMINA	0.79 (0.76–0.82)	0.79	0.82	0.95	0.20	0.47	−8.40
VINA	0.80 (0.77–0.84)	0.80	0.83	0.94	0.25	0.48	−8.20
AlphaFold model							
ADFR	0.80 (0.76–0.83)	0.78	0.82	0.95	0.25	0.51	−8.70
DOCK	0.72 (0.68–0.75)	0.73	0.75	0.95	0.05	0.4	−61.08
GNINA	0.75 (0.72–0.78)	0.79	0.81	0.96	0.15	0.43	−7.57
	0.82 (0.66–0.97)	0.87	0.94	0.77	0.95	0.70	−8.14
CNN affinity+	0.86 (0.84–0.88)	0.83	0.87	0.94	0.46	0.40	6.72
	0.94 (0.87–1) †	0.89†	0.95†	0.81†	0.96†	0.74†	7.05†
JDOCK	0.78 (0.76–0.82)	0.79	0.82	0.95	0.15	0.49	−8.12
PLANTS	0.86 (0.84–0.88)	0.80	0.84	0.91	0.47	0.59	−90.82
RXDOCK	0.73 (0.70–0.76)	0.60	0.69	0.73	0.57	0.41	−16.15
SMINA	0.78 (0.75–0.82)	0.78	0.80	0.95	0.14	0.49	−8.10
VINA	0.80 (0.77–0.83)	0.79	0.82	0.95	0.22	0.50	−8.30
1T9R							
ADFR	0.80 (0.76–0.82)	0.78	0.81	0.94	0.23	0.48	−8.60
DOCK	0.68 (0.66–0.72)	0.72	0.74	0.97	0.00	0.40	−44.25
GNINA	0.64 (0.57–0.65)	0.79	0.79	0.99	0.00	0.31	−5.64
CNN affinity+	0.76 (0.72–0.78)	0.79	0.80	0.98	0.80	0.43	5.47
JDOCK	0.67 (0.63–0.71)	0.80	0.80	0.99	0.03	0.26	−7.26
PLANTS	0.80 (0.75–0.81)	0.75	0.78	0.93	0.18	0.53	−81.61
RXDOCK	ND	ND	ND	ND	ND	ND	ND
SMINA	0.68 (0.62–0.70)	0.79	0.79	0.99	0.01	0.31	−7.30
VINA	0.65 (0.61–0.69)	0.79	0.79	0.99	0.02	0.26	−7.10

* Indicates ranking after Cartesian minimization. + indicates ranking using CNNaffinity. † Indicates results ranked by affinity after selecting results with a CNNscore ≥0.9.

The heuristic described in the previous section, which incorporated a CNN score threshold of ≥0.9 before affinity ranking, was used to produce another four ROC analyses, two for 1T9S, and two for the AlphaFold model, using only the results that passed the quality threshold. The use of this threshold produced a large increase in specificity (0.15–0.97 and 0.53–0.92 for 1T9S; 0.15–0.95 and 0.46–0.96 for the AlphaFold model) along with a small decrease in sensitivity (0.95–0.92 and 0.95–0.92 for 1T9S; 0.94–0.81 and 0.94–0.81 for the AlphaFold model) with GNINA affinity and CNN affinity. Youden’s index and the AUC also increased. This heuristic, based on the CNN score, could not be used for 1T9R because no docking results passed the quality threshold. These results highlight that docking affinities alone are poor classifiers that can be supplemented with information about quality, such as the CNN score.

### Structural visualization of sildenafil docking into PDE5A

**FIGURE 3:**
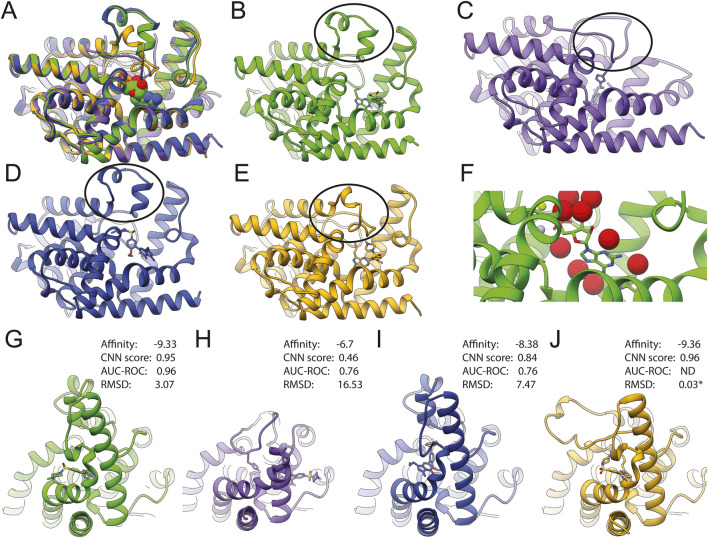
Structure comparison of PDE5A results with docked sildenafil (1T9S in green **(B, G)**; 1T9R in purple **(C, H)**, AlphaFold in deep blue **(D,I)**, and the model crystallized with sildenafil (2H42 in orange **(E,J)**. **(A)** Shows the superposition of all four structures. **(B–E)** show each model in the same orientation as in **A**, with an oval emphasizing the H-loop (residues L672-H685 in wild type). Residues 658–671 are missing on 1T9R, and residues 658–681 are chimeric in 1T9S (oval). **(G–J)** Display side views to emphasize that the binding location and pose for sildenafil were different between models. **(F)** Close-up of GMP bound to 1T9S, with water molecules in red, zinc in gray, and magnesium in green. * Indicates RMSD to XRD structure after CNN rescoring.

Superposition of the structures evaluated here (1T9S, 1T9R, and the AlphaFold model) with a counterpart crystallized with sildenafil (2H42) shows conformational changes in PDE5A’s H-loop (Figure 3, black ovals) that explain ligand accessibility. The H-loop is extended in the wild-type sildenafil-bound 2H42 (orange) (Figures 3E,J). In 1T9S (green), this region forms two small helices away from the catalytic site (Figures 3B,G), allowing ligand access; while in 1T9R (purple), histidine 678 on the extended H-loop seems to block ligand access (Figures 3C,H). With GNINA, sildenafil docked into 1T9S in a pose remarkably like the XRD determined (Figure 3G vs. J), with a resulting RMSD of 1.04 Å when excluding the ethylpiperazine motif (Figure 2, red square) and 3.07 Å when including it. This motif is stabilized by the H-loop in 2H24 but is solvent exposed in our docking to 1T9S. PDE5A contains numerous water molecules, magnesium, and zinc trapped in the binding site (Figure 3F). Their removal was compatible with successful docking with GNINA, aided by the CNN score, and produced a sildenafil pose very close to the crystallographic. The AlphaFold model has an accessible binding site (Figure 3D) like 1T9S and docks sildenafil in a similar site (Figure 3I), yet docking results were better for 1T9S. In turn, docking into 1T9R happens at a different site (Figure 3H). By comparing the parameters displayed in Figures 3G–J, we propose that a high CNN score identifies correct poses for potential binders that have high affinity and low RMSD (Figures 3G–J).

## Discussion

Docking methods are widely used to quickly screen molecular interactions with reasonable accuracy. Software availability and computer power allow the implementation of a docking campaign in any lab, yet many efforts lack quality parameters, which are not standardized in the field. The user is then left with a result of unclear relevance. The PDE5A-sildenafil interaction explored here exemplifies that two conditions are crucial to achieve relevant docking results: (1) careful receptor selection to ensure an accessible binding site and (2) a reliable quality metric to evaluate the results. We found that the most reliable quality metric was the CNN score from GNINA, which identified sildenafil at rank 26 and vardenafil at 52. Thus, using docking-calculated binding affinities alone is not accurate enough for correct ranking.

The CNN score aided receptor selection (Figure 1D), docking ranking (Figure 2), and ROC modeling (Table 1). Only GNINA provides this CNN quality metric. We propose that both result ranking and ROC must be performed for any given target to select good drug candidates. Still, the user must be aware of the limitations of the docking programs, in particular their low specificity, which entails that false negatives may be included in the top ranked. The selection of results by CNN score significantly improves the quality of the results. The real-world consequence is that true positives were correctly identified within the top-100 ranked molecules of more than 2,000 FDA-approved drugs when a target with a viable binding site was used. In the context of drug screening, it is crucial to consider that relevant docking results will not be only the few with the best affinity but may be in the top 5–7.5% as ranked by affinity, even after quality evaluations. This represents 100 or more ligands to consider for further testing, which is reasonable for most laboratories.

In our study, GNINA performed better than other programs in identifying true ligands in a pool of drugs. Thus, GNINA represents a great advance in docking, mostly because of its CNN score. It has been previously shown to work better than Vina (Sunseri and Koes, 2021) on 76% of the targets in the Database of Useful Decoys-Enhanced (DUD-E) and LIT-PCBA. GNINA captures solvent contributions to binding implicitly (Hochuli et al., 2018). In the current work, it increased specificity by reducing false negatives. These characteristics are likely due to the robustness of its CNN scoring model as well as the curated datasets used for its training (Francoeur et al., 2020), designed to avoid bias due to size. At present, docking is not good at identifying transient interactions and cryptic binding sites, and extensive ligand conformation sampling cannot be performed. These challenges may be tackled with stable diffusion by generating different conformations of the receptor and ligand. GNINA should be tested against more receptors, particularly those with bad AUC already described in the literature (Brozell et al., 2012; Allen et al., 2015).

Structural visualization of docked models should be the last and most dispensable step because it is harder to systematize and is subject to human biases. If a “less than 2 Å” metric had been used to detect binders in our model, even the best sildenafil result would have been discarded as a poor binder, highlighting that this metric is arbitrary.

## Conclusion

The use of GNINA calculated affinity and CNN score improved docking for repurposing.

We propose the following heuristic for all docking efforts using any program:

1. An XRD structure crystalized with a ligand is better than an AlphaFold model as a docking receptor.
2. Use GNINA to select a receptor that docks against its ligand with a ≥0.9 CNN score. Otherwise, use the receptor with the highest CNN score.
3. If the CNN score is not available, create a ROC analysis to identify the overall quality of the target, distinguish true vs. false binders, and select the most suitable docking program. This requires known true positives.
4. Consider the top 50 or 100 ligands ranked by affinity as potential binders to evaluate further.
5. Structure comparison and visualization should be the last step after all quality controls have been exercised, and it is not crucial to detect a true ligand.

As shown here, our heuristic improves the overall docking performance when using GNINA by taking advantage of its CNN score. The combination of *in silico* and/or *in vitro* techniques, in addition to the use of approved drugs, brings certainty to the results. Ultimately, all docking programs will suffer from bias given the data they were trained on; even affinities taken as correct can be a source of error (Landrum and Riniker, 2024).

Because docking with GNINA is fast and can be done on a desktop computer, we hope the scientific community will begin to use the CNN score to test whether our results can be generalized to any receptor.

Finally, conclusions from every experiment should be interpreted based on the technique limitations and in the context of biochemical, cellular, and organism-level information.

## Data Availability

The raw data supporting the conclusions of this article will be made available by the authors, without undue reservation.
