# Supplementary material for: Quality over quantity: how to get the best results when using docking for repurposing
Source: Front Bioinform. 2025 May 26;5:1536504. doi: 10.3389/fbinf.2025.1536504 (PMC12146287; doi:10.3389/fbinf.2025.1536504)
Supplement: Supplementary file 1 [file Table1.docx]

Command lines:

ADFR

adfr -l $f -t receptor.trg --jobName big --nbRuns 50 --maxEvals 25000000 -O -T

PLANTS

PLANTS1.2_64bit --mode screen plantsconfig

Vina

vina --config conf.txt --ligand $f --out ${b}/${b}.pdbqt --log ${b}/log.txt

Gnina

gnina -r receptor.pdbqt -l $f --center_x 15.88 --center_y 15.96 --center_z -28.7 --size_x 31.5 --size_y 32.25 --size_z 33 --out $f.gz --log $f.log --exhaustiveness 50 --num_modes 10

Smina

smina.static -r 1T9S.pdbqt -l $f --center_x 15.88 --center_y 15.96 --center_z -28.7 --size_x 31.5 --size_y 32.25 --size_z 33 --out $f.gz --log $f.log --exhaustiveness 50 --num_modes 10
