# Supplementary material for: Quality over quantity: how to get the best results when using docking for repurposing
Source: Front Bioinform. 2025 May 26;5:1536504. doi: 10.3389/fbinf.2025.1536504 (PMC12146287; doi:10.3389/fbinf.2025.1536504)
Supplement: Supplementary file 2 [file Table2.docx]

**Supplementary table 1.** File formats used by docking program and websites describing their preparation.

| Docking program | Receptor | Ligand | Reference |
| --- | --- | --- | --- |
| **ADFR** | pdbqt, trg | Pdbqt | https://ccsb.scripps.edu/adfr/documentation/ |
| **UCSF Dock** | mol2 | mol2 | https://ringo.ams.stonybrook.edu/index.php/DOCK_Tutorials |
| **Gnina** | pdbqt | pdbqt | https://github.com/gnina/gnina |
| **Jdock** | pdbqt | pdbqt | https://github.com/stcmz/jdock |
| **PLANTS** | mol2 | mol2 | https://github.com/purnawanpp/plants |
| **RxDock** | mol2 | sdf | https://rxdock.gitlab.io/ |
| **Smina** | pdbqt | pdbqt | https://github.com/mwojcikowski/smina |
| **Vina** | pdbqt | pdbqt | https://autodock-vina.readthedocs.io/en/latest/ |
