# Supplementary material for: Quality over quantity: how to get the best results when using docking for repurposing
Source: Front Bioinform. 2025 May 26;5:1536504. doi: 10.3389/fbinf.2025.1536504 (PMC12146287; doi:10.3389/fbinf.2025.1536504)
Supplement: Supplementary file 3 [file DataSheet1.pdf]

Suplementary Figure 1

AlphaFold

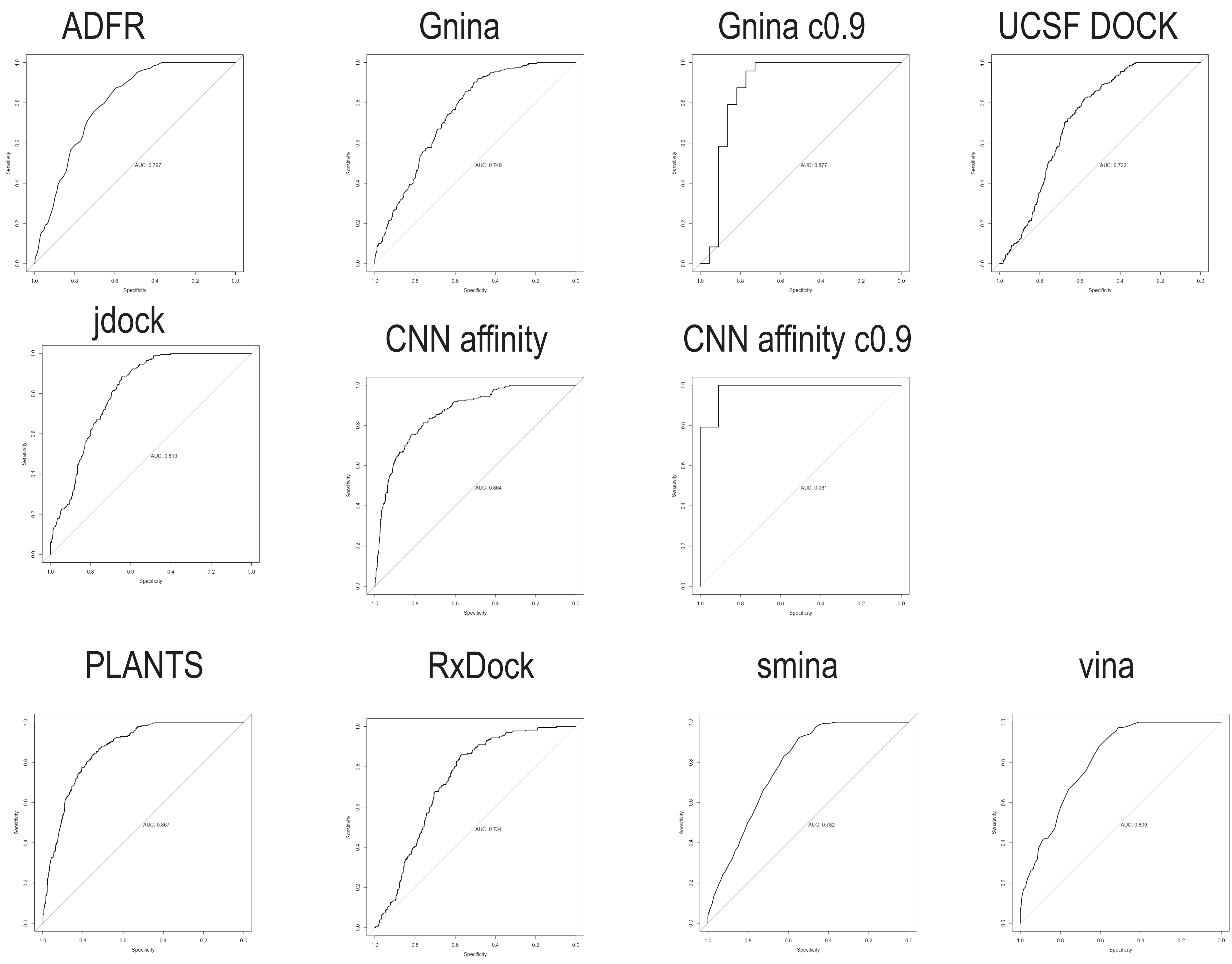

1T9R

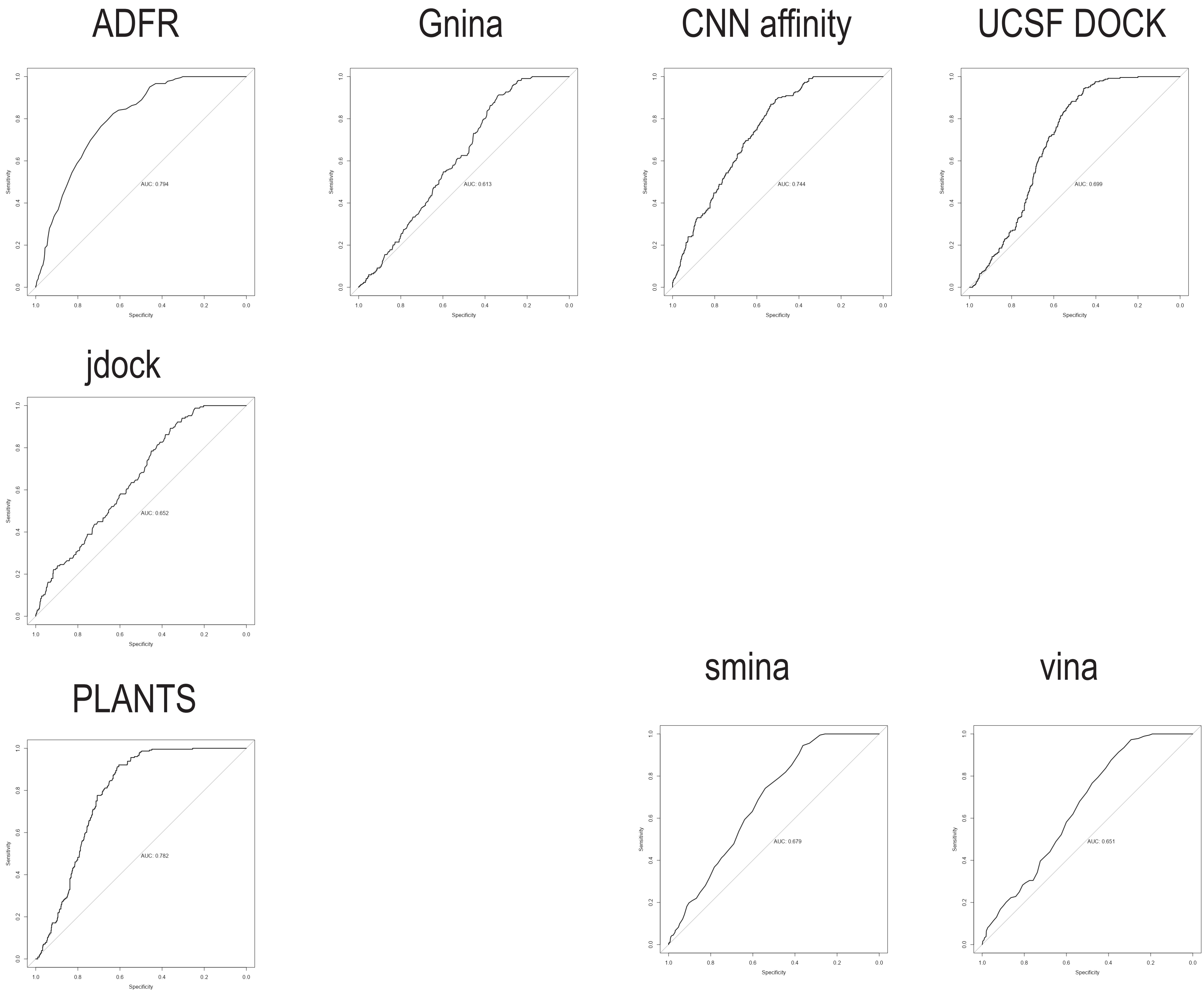
ADFR
AUC: 0.794
Gnina
AUC: 0.613
CNN affinity
AUC: 0.744
UCSF DOCK
AUC: 0.699
jdock
AUC: 0.652
PLANTS
AUC: 0.782
smina
AUC: 0.679
vina
AUC: 0.651
Sensitivity
Specificity
0.0
0.2
0.4
0.6
0.8
1.0

1T9S

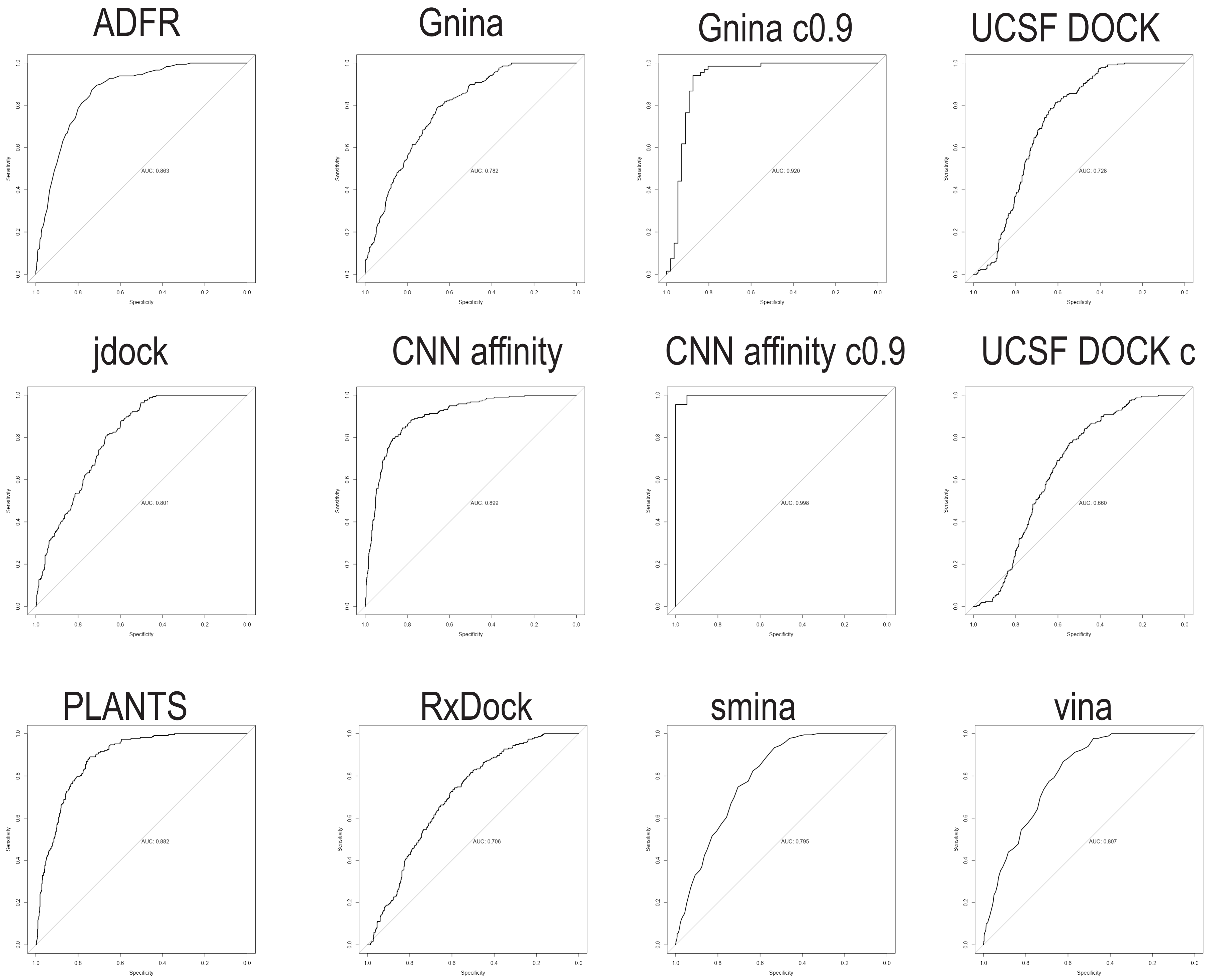

ADFR
AUC: 0.863
Gnina
AUC: 0.782
Gnina c0.9
AUC: 0.920
UCSF DOCK
AUC: 0.728
jdock
AUC: 0.801
CNN affinity
AUC: 0.899
CNN affinity c0.9
AUC: 0.998
UCSF DOCK c
AUC: 0.660
PLANTS
AUC: 0.882
RxDock
AUC: 0.706
smina
AUC: 0.795
vina
AUC: 0.807
Sensitivity
Specificity
