# Supplementary material for: Quality over quantity: how to get the best results when using docking for repurposing
Source: Front Bioinform. 2025 May 26;5:1536504. doi: 10.3389/fbinf.2025.1536504 (PMC12146287; doi:10.3389/fbinf.2025.1536504)
Supplement: Supplementary file 4 [file Table3.docx]

**Supplementary table 2.** Grid sizes used by each docking program.

| Docking program | Grid size (Å) |
| --- | --- |
| **ADFR** | 0.375 |
| **UCSF Dock** | 0.4 |
| **Gnina** | 0.375 |
| **Jdock** | 0.125 |
| **PLANTS** | 0.375 |
| **RxDock** | 0.5 |
| **Smina** | 0.375 |
| **Vina** | 0.375 |
