# Supplementary material for: Quality over quantity: how to get the best results when using docking for repurposing
Source: Front Bioinform. 2025 May 26;5:1536504. doi: 10.3389/fbinf.2025.1536504 (PMC12146287; doi:10.3389/fbinf.2025.1536504)
Supplement: Supplementary file 5 [file Table4.docx]

**Supplemental Table 3**. RMSD between Cα for the selected receptor structures.

| Structures  (RMSD, Å) | AF model | 1T9R | 1T9S | 2H42 |
| --- | --- | --- | --- | --- |
| AF model | 0 | 3.6 | 1.2 | 3.8 |
| 1T9R |  | 0 | 3.5 | 3.9 |
| 1T9S |  |  | 0 | 3.7 |
| 2H42 |  |  |  | 0 |
